# Fine-Tuned Large Language Model for Extracting Pretreatment Pancreatic Cancer According to Computed Tomography Radiology Reports

**DOI:** 10.1007/s10278-025-01637-w

**Published:** 2025-08-15

**Authors:** Hiroshi Hirakawa, Koichiro Yasaka, Takuto Nomura, Rin Tsujimoto, Yuki Sonoda, Shigeru Kiryu, Osamu Abe

**Affiliations:** 1 Department of Radiology, Graduate School of Medicine, The University of Tokyo, 7-3-1 Hongo, Bunkyo-Ku, Tokyo, 113-8655 Japan; 2 Department of Radiology, International University of Health and Welfare Narita Hospital, 852 Hatakeda, Narita, Chiba 286-0124 Japan

**Keywords:** Pancreatic cancer, Natural language processing, CT radiology reports

## Abstract

This study aimed to examine the performance of a fine-tuned large language model (LLM) in extracting pretreatment pancreatic cancer according to computed tomography (CT) radiology reports and to compare it with that of readers. This retrospective study included 2690, 886, and 378 CT reports for the training, validation, and test datasets, respectively. Clinical indication, image finding, and imaging diagnosis sections of the radiology report (used as input data) were reviewed and categorized into groups 0 (no pancreatic cancer), 1 (after treatment for pancreatic cancer), and 2 (pretreatment pancreatic cancer present) (used as reference data). A pre-trained Bidirectional Encoder Representation from the Transformers Japanese model was fine-tuned with the training and validation dataset. Group 1 data were undersampled and group 2 data were oversampled in the training dataset due to group imbalance. The best-performing model from the validation set was subsequently assessed using the test dataset for testing purposes. Additionally, three readers (readers 1, 2, and 3) were involved in classifying reports within the test dataset. The fine-tuned LLM and readers 1, 2, and 3 demonstrated an overall accuracy of 0.942, 0.984, 0.979, and 0.947; sensitivity for differentiating groups 0/1/2 of 0.944/0.960/0.921, 0.976/1.000/0.976, 0.984/0.984/0.968, and 1.000/1.000/0.841; and total time required for classification of 49 s, 2689 s, 3496 s, and 4887 s, respectively. Fine-tuned LLM effectively extracted patients with pretreatment pancreatic cancer according to CT radiology reports, and its performance was comparable to that of readers in a shorter time.

## Introduction

Pancreatic cancer is one of the leading causes of cancer-related deaths with a 5-year survival rate of approximately 5% [1]. It is the sixth leading cause of cancer-related death globally [2]. The difficulty of early detection of pancreatic cancer is one reason for its poor treatment outcome. Pancreatic cancer is usually diagnosed at a late stage when symptoms become apparent due to the special anatomical location of the pancreas. Most patients with pancreatic cancer present with locally advanced or distant metastatic disease (80–85%), and only a minority of patients are eligible for surgical resection (15–20%) [3, 4]. Therefore, pancreatic cancer requires appropriate clinical management, especially when diagnosing it at an early stage.

Computed tomography (CT) is the first-line imaging modality and has been utilized for suspected pancreatic cancer [5]. Effectively using vast CT data plays a crucial role in improving patient care and supporting research. Developing an automated system to extract untreated pancreatic cancer reports serves as a fail-safe system, preventing radiologists, especially with fewer years of experience, from forgetting to alert clinicians when they detect untreated pancreatic cancer. Moreover, it would support research by enabling efficient patient cohort formation from a significant number of cases. For example, extracting untreated pancreatic cancer cases and examining their imaging characteristics may provide useful findings for early detection. Furthermore, it is also useful when young radiologists learn about the imaging findings of pancreatic cancer separately before and after treatment. However, automatically analyzing unstructured radiology reports is challenging [6].

Applications of deep learning to radiology have been gaining wide attention since the mid-2010s [7, 8]. Large language modes (LLMs), such as generative pre-trained transformers (GPTs) and Bidirectional Encoder Representations from Transformers (BERTs), have attracted significant attention in recent years. GPT-4 demonstrates high performance in establishing pancreatic ductal adenocarcinoma synoptic reports from original reports and categorizing tumor resectability [9]. However, the majority of established LLMs require input data to be uploaded to the server via the Internet, which raises privacy concerns [10]. Consequently, demand for locally run models is increasing. Some pre-trained LLMs can be downloaded to local computers and can be fine-tuned with data from the fields of radiology to obtain appropriate output in a short time [11–16]. Therefore, we hypothesized that LLMs can be fine-tuned to efficiently extract patients with untreated pancreatic cancer according to CT radiology reports with high accuracy in a short time.

This study aimed to investigate the performance of fine-tuned LLM in extracting CT reports of patients with pretreatment pancreatic cancer according to CT radiology reports and to compare its performance with that of readers with fewer years of experience.

## Materials and Methods

This retrospective study obtained approval from our Institutional Review Board, waiving the requirement for obtaining written informed consent from patients due to the retrospective study design.

### Patients

**Fig. 1:**
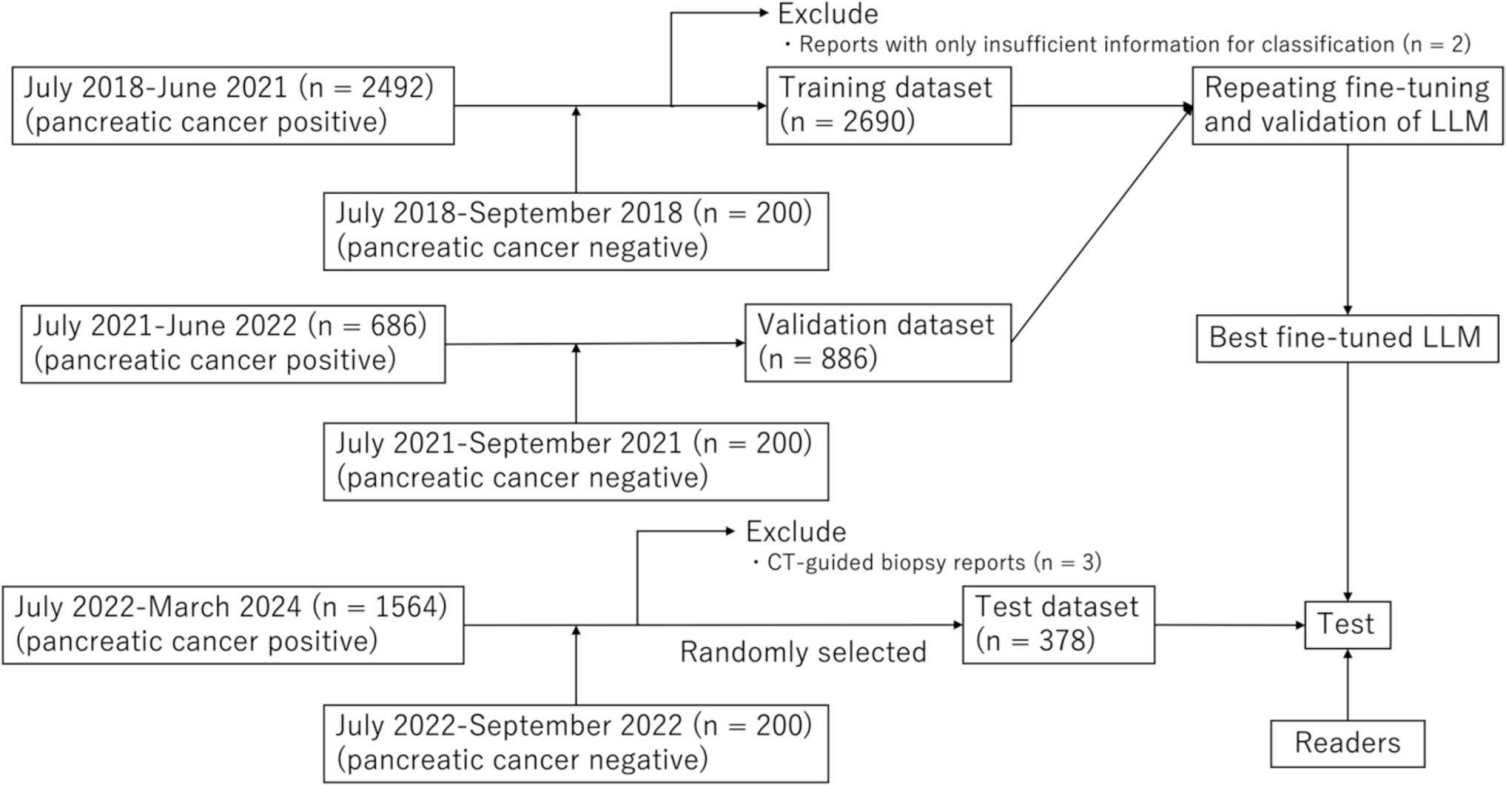
Data extraction process and schematic for training and evaluation. LLM, large language model

A radiologist (radiologist A), with a 3-year imaging experience, searched medical image management and processing systems for patients whose CT radiological report contained the term “pancreatic cancer.” The training, validation, and test datasets included patients who underwent CT from July 2018 to June 2021, from July 2021 to June 2022, and from July 2022 to March 2024, respectively. The reports of the first 200 cases of CT performed in July-September 2018, July-September 2021, and July-September 2022 were also extracted and included in the training, validation, and test datasets, respectively, to add reports without the word “pancreatic cancer” to these datasets. We excluded two and three reports from the training and test datasets, respectively, which contained insufficient information for classification or were CT-guided biopsy reports. To protect privacy, patients’ names and IDs are not included in these datasets. To facilitate fair evaluation of model performance across all groups and to reduce the burden on human readers during manual annotation, the test dataset was artificially balanced to include equal number of cases in each group, with random selection of 126 reports per group. Finally, the training, validation, and test datasets included 2690, 886, and 378 patients, respectively (Fig. 1). Their radiological reports, encompassing the clinical indication section, image finding section, and imaging diagnosis section, were extracted in CSV format. They were concatenated in that order for generating input data. Radiologists, with at least 5 years of imaging experience, have written all radiological reports in Japanese.

### Reference Standard

The clinical indication, image finding, and imaging diagnosis sections of the radiological report were reviewed, and the report was categorized into groups 0 (patient without pancreatic cancer), 1 (patient after pancreatic cancer treatment or patient who was already diagnosed with pancreatic cancer), and 2 (patient with pretreatment pancreatic cancer or patient who were suspected to have pretreatment pancreatic cancer). Radiologist A performed these classifications for the training, validation, and test datasets.

### Fine-Tuning of the Pre-trained LLM

**Fig. 2:**
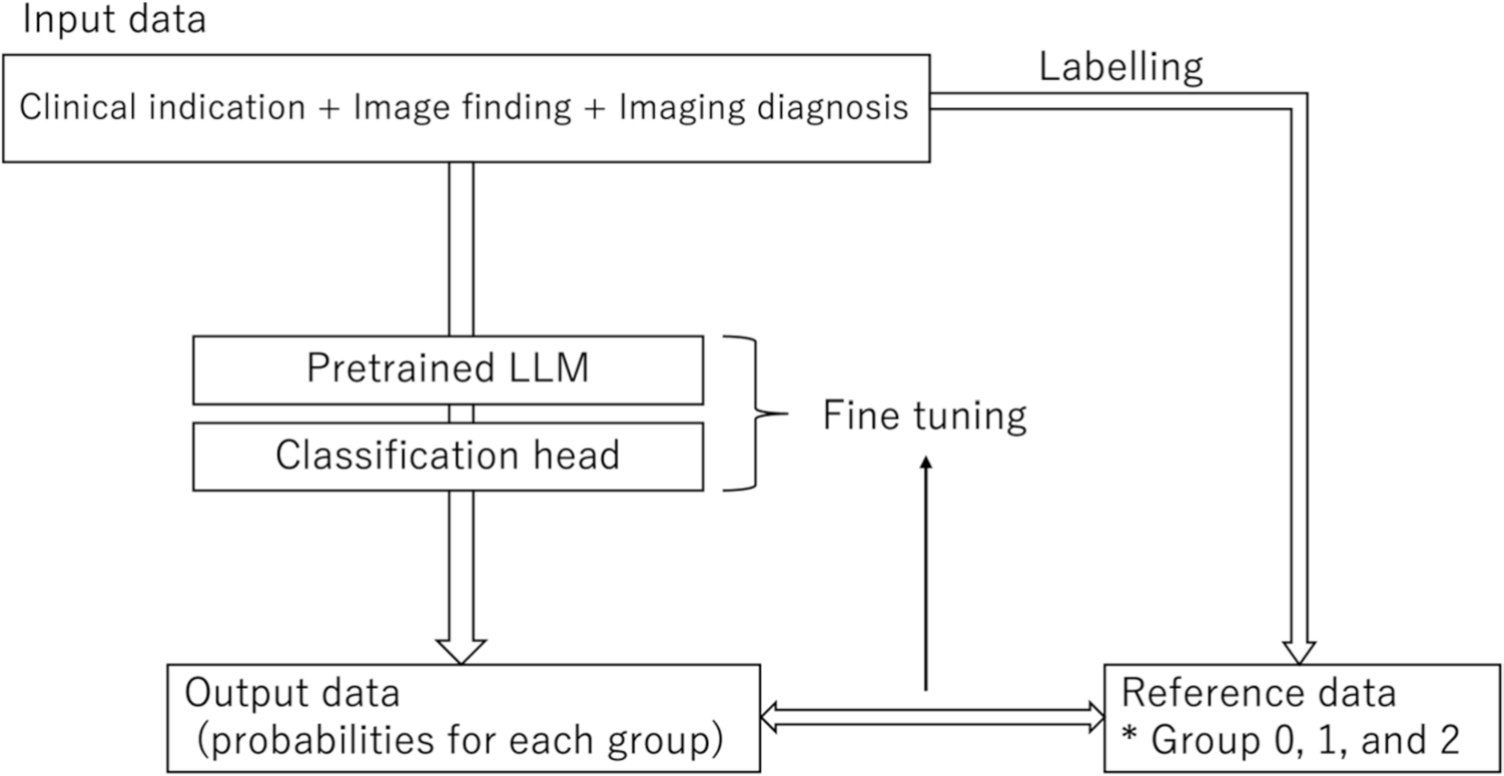
Algorithm of fine-tuning LLM for report classification. LLM, large language model

The pre-trained Bidirectional Encoder Representations from the Transformers Japanese model (https://huggingface.co/cl-tohoku/bert-base-japanese) were fine-tuned using the programming language of Python version 3.10.13 (https://www.python.org/) and the Transformers library version 4.35.2 (https://huggingface.co/) on a workstation equipped with a central processing unit of Core™ i9-12900F (Intel), a graphic processing unit of GeForce RTX 3090 (NVIDIA), and a 128-GB RAM. The model, consisting of 12 layers, 768 hidden state dimensions, and 12 attention heads, had been pre-trained with Japanese Wikipedia as of September 1, 2019. The AutoModelForSequenceClassification class method in the Transformers library was used to set the model to categorize reports, consisting of the concatenated sentences of the clinical indication, image finding section, and imaging diagnosis section, into three groups based on the logits for each group (Fig. 2). We identified the number of epochs for the fine-tuning process and set it at 10 according to the model’s performance on the training and validation datasets. Other hyperparameters were set to the default values of the Transformers library (learning rate, 5e − 5; batch size, 8).

Fine-tuning and validation were conducted (session 1) using the training and validation dataset. For privacy reasons, pre-trained LLM was downloaded to a local computer and patient data were not uploaded to the server via the Internet. In addition, only the texts of the reports were used, and the CT images in digital imaging and communications in medicine format were not included in this learning process. Undersampling with or without oversampling was performed in sessions 2 and 3 to assess the effect of balancing the number of patients across groups. Concretely, only undersampling of group 1 data (500 out of 2025 patients were randomly selected) was conducted in session 2. Undersampling of group 1 data (500 out of 2025 patients were randomly selected) and oversampling of group 2 data were performed in session 3. Oversampling of group 2 was conducted by duplicating each report once, effectively doubling the number of group 2 samples. The oversampled versions were generated by swapping the positions of the clinical indication and imaging diagnosis sections. No additional text augmentation techniques such as synonym substitution, paraphrasing, or sentence-level perturbation were used. This simple augmentation aimed to introduce structural variation while preserving the original content and clinical meaning. Fine-tuning and validation were conducted thrice in each session. In each session, the median of the required time, accuracy, and sensitivity for each group were calculated.

We utilized the sensitivity for group 2 in selecting the best performance model in the validation dataset as a key metric because the sensitivity for this group is more important than that of groups 0 and 1. A model developed in session 3 demonstrated the best sensitivity for group 2. Therefore, we conducted fine-tuning and validation 12 more times (training and validation were performed 15 times in total for session 3). The code utilized for the fine-tuning is available upon a reasonable request.

### Test Phase of the Fine-Tuned LLM

The model with the best performance in the validation dataset was subsequently evaluated in the independent test dataset. The same computer was used as in the fine-tuning of the pre-trained LLM. Three readers (readers 1, 2, and 3 with 2-, 1-, and 0-year imaging experience, and with 4-, 3-, and 2-year clinical experience, respectively) participated in the manual classification of the reports in the test dataset into three groups to compare the performance of LLM to that of readers. The categorized group data and the time required to complete all the tasks were documented.

### Statistical Analyses

Statistical analyses were conducted with EZR (Saitama Medical Center, Jichi Medical University, Saitama, Japan) [17], which is a graphical user interface for R. The McNemar test was utilized to compare the sensitivity and specificity for each group and the overall accuracy in the test dataset between the fine-tuned LLM and the readers. The fine-tuned LLM’s diagnostic performance in differentiating group 2 from the other groups was assessed by calculating the area under the receiver operating characteristic curve (AUC) according to the probability for group 2 calculated from the logit data. The Bonferroni correction was applied and the statistical significance was set at a *p*-value of 0.017 (= 0.050/3) because of the multiple comparisons.

## Results

### Patients

Table 1 shows the background information on the patients. The numbers of patients in groups 0/1/2 were 443/2025/222, 245/560/81, and 126/126/126 for the training, validation, and test datasets, respectively.

**Table 1:** Patient background

	Training	Validation	Test
Number of patients	2690	886	378
Age (years)	69.2 ± 11.4	68.4 ± 13.6	68.0 ± 13.8
Sex (male/female)	1702/988	539/347	232/146
Number of radiologists who confirmed report	23	16	21
Number of patients in each group			
0: Pancreatic cancer absent	443	245	126
1: Posttreatment pancreatic cancer	2025	560	126
2: Pretreatment pancreatic cancer	222	81	126

### Effect of Undersampling and Oversampling on the Sensitivity of Each Group in the Validation Dataset

Table 2 summarizes the results of the fine-tuning and validation in sessions 1–3. The median time required for the training in sessions 1, 2, and 3 was 986 s, 451 s, and 497 s, and the median accuracy was 0.962, 0.966, and 0.962, respectively. The median sensitivity for groups 0/1/2 was 0.939/0.991/0.803, 0.963/0.977/0.889, and 0.951/0.973/0.913 in sessions 1–3, respectively. The median sensitivity for group 2 was the highest in session 3; thus, fine-tuning and validation were added and conducted 15 times in total in session 3. The best model in session 3 was selected for further assessment of its performance in the test dataset.

**Table 2:** Result of fine-tuning and validation in sessions 1–3

	Accuracy	Sensitivity for each group			Time required (s)
		group 0	group 1	group 2	
Session 1					
A	0.956 (847/886)	0.939 (230/245)	0.986 (552/560)	0.803 (65/81)	986
B	0.963 (853/886)	0.955 (234/245)	0.991 (555/560)	0.790 (64/81)	984
C	0.962 (852/886)	0.931 (228/245)	0.991 (555/560)	0.852 (69/81)	1008
Session 2 (undersampling of group 1)					
A	0.968 (856/886)	0.963 (236/245)	0.979 (548/560)	0.914 (74/81)	453
B	0.966 (121/886)	0.971 (238/245)	0.975 (546/560)	0.889 (72/81)	450
C	0.958 (849/886)	0.959 (235/245)	0.977 (547/560)	0.827 (67/81)	451
Session 3 (undersampling of group 1 and oversampling of group 2)					
A	0.957 (848/886)	0.947 (232/245)	0.968 (542/560)	0.913 (74/81)	497
B	0.962 (852/886)	0.951 (233/245)	0.975 (546/560)	0.901 (73/81)	497
C	0.970 (859/886)	0.967 (238/245)	0.973 (545/560)	0.951 (77/81)	498

### Performance of the Fine-Tuned LLM and Readers in the Test Dataset

Table 3 shows a confusion matrix comparing the reference standard to the prediction data by the best-fine-tuned LLM and readers. Table 4 presents the accuracy, sensitivity, and specificity data. The accuracy was lower in the fine-tuned LLM (0.942) than in readers 1 (0.984) and 2 (0.979) and was comparable to reader 3 (0.947). The sensitivity in differentiating groups 0 and 1 was slightly lower in the fine-tuned LLM (0.944 and 0.960, respectively) than in the readers (0.976–1.000 and 0.984–1.000, respectively). However, no statistically significant differences were observed. The sensitivity in differentiating group 2 was comparable between the fine-tuned LLM (0.921) and readers (0.841–0.976). The specificity of group 2 was comparable between the fine-tuned LLM (0.984) and readers (0.992–1.000).

**Table 3:** Confusion matrix for a reference standard versus prediction data in the test dataset

	Reference standard		
	Group 0 (*n* = 126)	Group 1 (*n* = 126)	Group 2 (*n* = 126)
Large language model			
Group 0 (*n* = 129)	119	4	6
Group 1 (*n* = 129)	4	121	4
Group 2 (*n* = 120)	3	1	116
Reader 1			
Group 0 (*n* = 126)	123	0	3
Group 1 (*n* = 129)	3	126	0
Group 2 (*n* = 123)	0	0	123
Reader 2			
Group 0 (*n* = 128)	124	2	2
Group 1 (*n* = 126)	0	124	2
Group 2 (*n* = 124)	2	0	122
Reader 3			
Group 0 (*n* = 133)	126	0	7
Group 1 (*n* = 139)	0	126	13
Group 2 (*n* = 106)	0	0	106

**Table 4:** Data for sensitivity, specificity, and time required in the test dataset

	Fine-tuned LLM	Reader 1		Reader 2		Reader 3	
		Score	Comparison	Score	Comparison	Score	Comparison
Accuracy	0.942 (356/378)	0.984 (372/378)	0.002*	0.979 (370/378)	0.011*	0.947 (358/378)	0.860
Sensitivity for each group							
Group 0	0.944 (119/126) [0.889, 0.997]	0.976 (123/126) [0.932, 0.995]	0.343	0.984 (124/126) [0.944, 0.998]	0.182	1.000 (126/126) [0.944, 0.989]	N/A
Group 1	0.960 (121/126) [0.910, 0.987]	1.000 (126/126) [0.957, 1.000]	N/A	0.984 (124/126) [0.944, 0.998]	0.371	1.000 (126/126) [0.957, 1.000]	N/A
Group 2	0.921 (116/126) [0.859, 0.961]	0.976 (123/126) [0.932, 0.995]	0.046	0.968 (122/126) [0.921, 0.991]	0.149	0.841 (106/126) [0.766, 0.900]	0.044
Specificity for each group							
Group 0	0.960 (242/252) [0.928, 0.981]	0.988 (249/252) [0.966, 0.998]	0.046	0.984 (248/252) [0.960, 0.996]	0.114	0.972 (245/252) [0.944, 0.989]	0.579
Group 1	0.968 (244/252) [0.938, 0.986]	0.988 (249/252) [0.966, 0.998]	0.228	0.992 (250/252) [0.972, 0.999]	0.114	0.948 (239/252) [0.913, 0.972]	0.383
Group 2	0.984 (248/252) [0.960, 0.996]	1.000 (252/252) [0.978, 1.000]	N/A	0.992 (250/252) [0.972, 0.999]	0.683	1.000 (252/252) [0.978, 1.000]	N/A
Time required (s)	49	2689		3496		4887	

Comparisons between fine-tuned LLM against readers with the McNemar test. In brackets, 95% confidence intervals are provided

*LLM* large language model, *N/A* not applicable

*p < 0.017

**Fig. 3:**
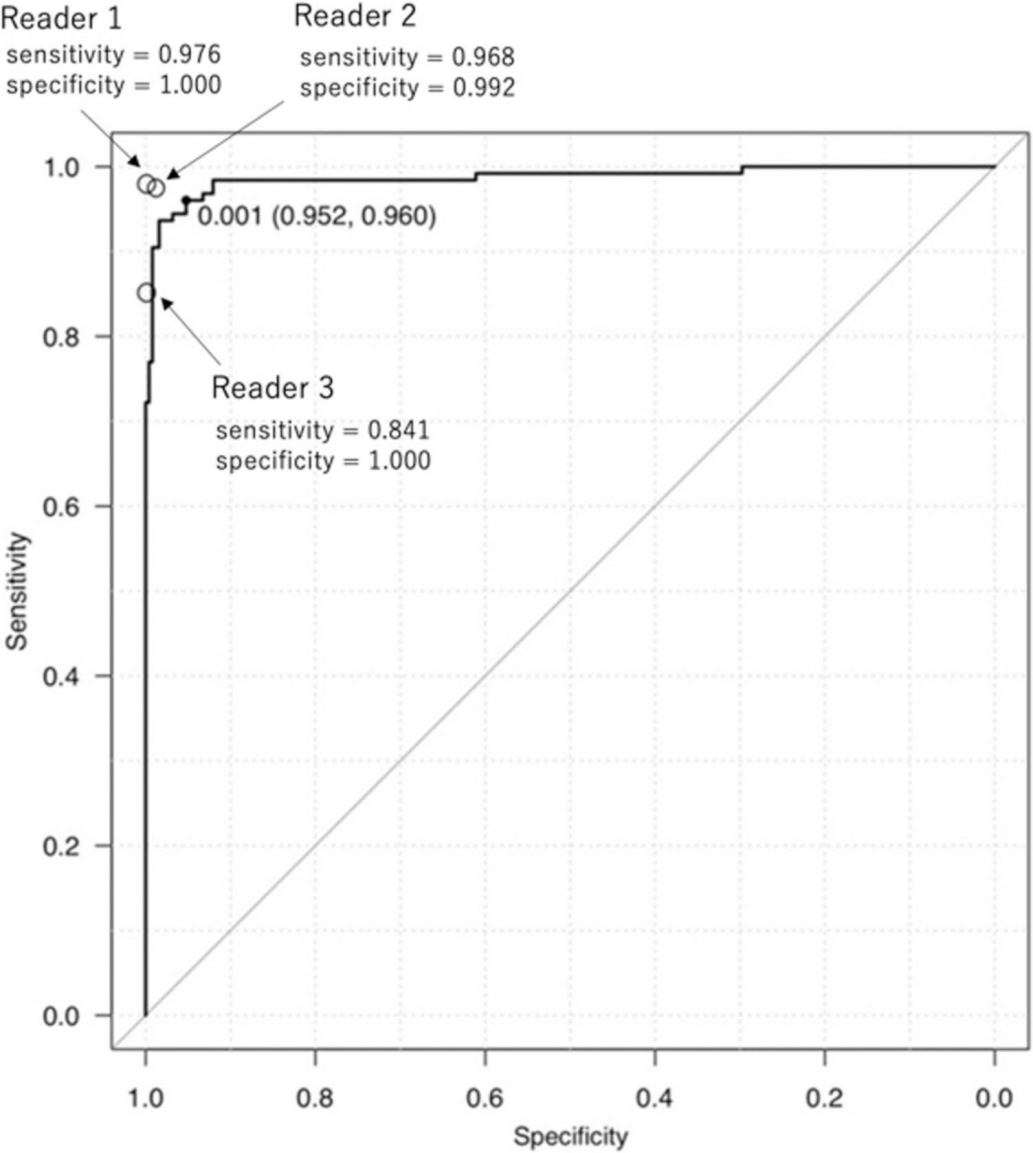
Receiver operating characteristic curve for the fine-tuned LLM in discriminating group 2 from other groups in the test dataset. The area under the receiver operating characteristic curve was 0.987. The radiologists’ performance was plotted as hollow circles. A black circle indicates the threshold (specificity, sensitivity) that achieves the Youden index

The diagnostic performance for discriminating group 2 from the other groups was high using the output probability for this group, with an AUC of 0.987 (95% confidence interval: 0.9741–1.000) (Fig. 3). The threshold (sensitivity and specificity) value which achieved the Youden index was 0.001 (0.960 and 0.952).

The fine-tuned LLM was used to categorize all reports in the test dataset within 49 s, which was 54.9-, 71.3-, and 99.7-fold faster than readers 1 (2689 s), 2 (3496 s), and 3 (4887 s), respectively.

## Discussion

Our study reveals that fine-tuned LLM can extract pretreatment pancreatic cancer reports (group 2) with high sensitivity and specificity comparable to human readers within a remarkably shorter time. This ability enables the LLM to serve as a fail-safe system to prevent radiologists from failing to alert clinicians when they incidentally detect pancreatic cancer, which may result in early therapeutic interventions. Our model can be operated on a local computer and there is no need to upload patient data to the Internet server. Therefore, security issues are unlikely to occur when operated within each facility.

There were statistically significant differences in the overall accuracy between LLM and readers 1 and 2, but LLM’s overall accuracy of 94.2% was still acceptable. No significant differences were found in sensitivity or specificity for any group. Although there were no statistically significant differences, the sensitivity of LLM for group 0 and 1 was slightly lower than that of readers. This was due to LLM’s misclassification of post-treatment or known pancreatic cancer as group 0, and of reports that did not contain the word “pancreatic cancer” as group 1 or 2. These mistakes were rarely seen in readers. The sensitivity of LLM for group 2 was relatively lower than that for the other groups, which was also observed in the readers’ results. This can be explained by the fact that group 2 contained not only apparent pancreatic cancer cases but also ambiguous cases in which the possibility of pancreatic cancer could not be ruled out. Ambiguities in radiology reports—such as phrases like “suspicious for pancreatic cancer,” “cannot rule out malignancy,” or “needs follow up”—may have contributed to the decreased sensitivity for group 2. These expressions lack definitive diagnostic labels and pose challenges even for human readers. To mitigate this, future model training could incorporate uncertainty-aware classification frameworks or leverage annotated corpora that label degrees of diagnostic confidence. Although the area under the ROC curve (AUC) for group 2 was high (0.987), the optimal threshold determined by the Youden index was 0.001. While this value was statistically optimal for maximizing the sum of sensitivity and specificity, such a low cutoff is likely impractical in clinical settings. This suggests that the model was conservative in assigning high probabilities for group 2, and that the threshold should be adjusted in advance based on clinical needs, or chosen using a practical method that balances sensitivity, specificity, and clinical priorities.

False negatives for group 2—patients with undetected pretreatment pancreatic cancer—carry significant clinical risk. Missed cases may delay surgical consultation or therapeutic intervention, leading to disease progression beyond the window for surgical resection. Therefore, maximizing sensitivity for group 2 is not only important for model performance but also critical from a clinical safety perspective. While models such as Chat GPT, which has recently received a lot of attention, may further improve LLM’s results, its cloud-based nature raises privacy concerns in clinical applications.

The overall 5-year survival rate for pancreatic cancer is reportedly 12.5%, but it rises to approximately 80% when detected early [18]. This indicates the importance of early detection of pancreatic cancer. Therefore, when pancreatic cancer is detected on CT, prompt management by physicians is essential. Announcing the presence of these patients is the role of radiologists, whereas establishing an alert system serves as a fail-safe system for them. However, extracting patients with untreated pancreatic cancer from the CT radiology report has not been a simple task due to the ambiguities of natural language. Keyword search with “pancreatic cancer” results in extracting not only patients with untreated pancreatic cancer but also those with posttreatment pancreatic cancer or those without pancreatic cancer. The developed system in this study, which demonstrated the sensitivity and specificity for untreated pancreatic cancer comparable to radiologists, can be a fail-safe system. For example, we can extract suspected cases of untreated pancreatic cancer from CT reports created in the last month and check whether they are appropriately followed up.

The fine-tuned LLM can categorize CT reports much faster than the readers. The required time for the model (0.130 s [= 49 s/378]) was comparable to those demonstrated in previous similar studies (0.148–0.348 s) [11–13]. Additionally, the ability to quickly categorize CT reports by presence and treatment status of pancreatic cancer would be useful for future research, making the extraction of eligible patients possible following their cancer status.

The imbalance between classes is generally associated with biased sensitivity across groups [19]. It leads to decreased sensitivity for the group with a small number. In this study, pretreatment pancreatic cancer (group 2) has fewer cases than posttreatment pancreatic cancer (group 1), which can reduce its sensitivity. However, this is problematic because the sensitivity for group 2 is of paramount importance. Our study indicated that undersampling group 1 cases and oversampling group 2 cases improved the sensitivity for group 2 without compromising the sensitivity for the other groups. Therefore, these methods seem to be effective for addressing class imbalance while maintaining generalizability.

Our study has some limitations. First, we utilized the BERT Japanese model as our LLM. The performance of the model varies across different languages; thus, our result is not necessarily applicable to other languages. Second, the model was fine-tuned and assessed with only the CT reports of a single institution, so it remains unclear whether the same level of performance would be achievable at other institutions. Temporal and geographic sets are classified as external datasets by Walston et al. [20]. While we attempted to enhance robustness by using temporally distinct datasets for training, validation, and test, this approach does not substitute for external geographical testing. Moreover, differences in institution practices and radiology reporting styles may reduce the model’s applicability without prior adaptation or retraining. Therefore, further validation using datasets from multiple institutions with varying reporting styles and clinical practices is desirable to confirm the generalizability and clinical applicability of our model. Third, the test dataset was artificially balanced to include an equal number of reports for each group. However, it does not reflect the real-world class distribution. As a result, performance metrics such as accuracy may be slightly inflated, and future evaluations using naturally imbalanced datasets might be necessary for a more realistic assessment of the model’s effectiveness. Fourth, the accuracy of the fine-tuned LLM was slightly lower than that of two readers. However, the sensitivity of the model for pretreatment pancreatic cancer, which was the key metric, was comparable. Fifth, the model was specifically designed for pancreatic cancer analysis and was limited to interpreting CT reports. Therefore, it cannot be applied to other diseases or modalities. Finally, this is a retrospective study. A future prospective study would be necessary to consolidate the findings of our study.

In conclusion, the fine-tuned LLM was useful in effectively extracting patients with pretreatment pancreatic cancer, and its performance was comparable to manual annotation by less-experienced readers in a significantly shorter time.

## Data Availability

Due to the nature of this research, patients of this study did not agree for their data to be shared publicly, so supporting data is not available.
